# *N*-Heterocyclic Carbene-Polyethyleneimine (PEI) Platinum Complexes Inducing Human Cancer Cell Death: Polymer Carrier Impact

**DOI:** 10.3390/ijms19113472

**Published:** 2018-11-05

**Authors:** May Wantz, Mathilde Bouché, Georges Dahm, Neïla Chekkat, Sylvie Fournel, Stéphane Bellemin-Laponnaz

**Affiliations:** 1 UMR 7199, Faculté de Pharmacie, Université de Strasbourg-CNRS, 74 Route du Rhin, BP 60024, 67401 Illkirch CEDEX, France; may.wantz@etu.unistra.fr (M.W.); Neila.Chekkat@sanofi.com (N.C.); 2 Institut de Physique et Chimie des Matériaux de Strasbourg, UMR 7504 CNRS-Université de Strasbourg, 23 rue du Loess, 67000 Strasbourg, France; mathilde.bouche9@gmail.com (M.B.); georges.dahm@etu.unistra.fr (G.D.)

**Keywords:** antitumoral activity, platinum, *N*-Heterocyclic carbene, polyethyleneimine

## Abstract

The high interest in *N*-Heterocyclic platinum carbene complexes in cancer research stems from their high cytotoxicity to human cancer cells, their stability, as well as their ease of functionalization. However, the development of these new molecules as anticancer agents still faces multiple challenges, in particular solubility in aqueous media. Here, we synthesized platinum-NHC bioconjugates that combine water-solubility and cytotoxicity by using polyethyleneimine as polymer carrier. We showed on 8 different types of cells that the activity of these conjugates is modulated by the size of the polymer and the overall density of metal ions onto polymer chains. Using HCT116 cells, the conjugates displayed an effective activity after only 45 min of exposure in vitro correlated with a quick uptake by the cells as shown by the use of various fluorescent-tagged derivatives.

## 1. Introduction

**Figure 1:**
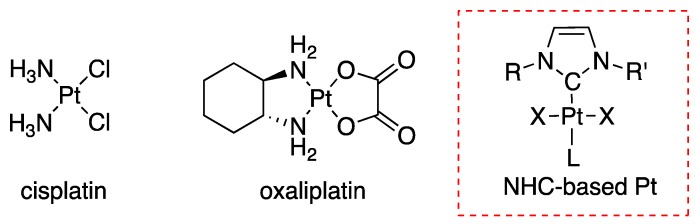
Chemical structures of platinum complexes cisplatin, oxaliplatin and *N*-Heterocyclic carbene platinum (II) complexes that showed high cytotoxic activities against cancer cells.

Due to their unique properties, metal ions are the subject of intensive research for the development of drugs against acute illness [1]. The interest in metal-based drugs has essentially started with the discovery of the antiproliferative activity of cisplatin by Rosenberg, which is still now the most widely used anticancer drug (Figure 1) [2]. However, cancer treatment with cisplatin (and its derivatives such as oxaliplatin) is accompanied by various severe toxic side effects and in addition tumour resistance is a real concern that pushes chemists and biologists to develop alternative molecules [3]. Nevertheless, the success of cisplatin remains a motivation for the development of new platinum complexes displaying lower side effects [4,5].

Transition metal complexes featuring *N*-Heterocyclic carbene (NHC) ligands have received much interest in recent years in the context of cancer therapy [6]. Positive preliminary results motivated research and development in this direction and recently many NHC-containing platinum compounds have shown high activity in vitro against various cancer cell lines with cytotoxicity significantly higher than cisplatin (Figure 1) [7,8,9]. However, the (pre)clinical development of these new molecules as anticancer agents still faces multiple challenges, including the clarification of their mode of action. Moreover, a major barrier of these promising candidates remains their poor solubility in physiologic medium. Thus, the development of innovative strategies to bring solubility in water while maintaining the biological activity is a topical subject for these systems.

We recently highlighted that polyethyleneimine (PEI), a polymer widely used as transfection agent, can be used as ligand for *N*-Heterocyclic carbene platinum complexes to generate NHC-Pt-PEI conjugates that induce human cancer cell death in vitro and in vivo [10]. Interestingly, we showed that a part of the cytotoxicity induced by our compounds was not affecting the nucleus and seemed related to mitochondrial dysfunction. Herein, we wish to report the synthesis and in vitro activities of such conjugates where the polymer type (linear or branched) and size (low Mw to high Mw) were varied. We also studied the local platinum concentration effect onto the biological activity and concluded that an optimal balance between all these parameters is required for an efficient anticancer activity. To go further in the understanding of the conjugate’s mode of action, we evaluated their cellular uptake using fluorescent derivatives and showed that a short exposure is sufficient to induce tumour cell cytotoxicity.

## 2. Results

### 2.1. Synthetic Procedure and Characterization

#### 2.1.1. NHC-Pt-PEI Conjugate

**Figure 2:**
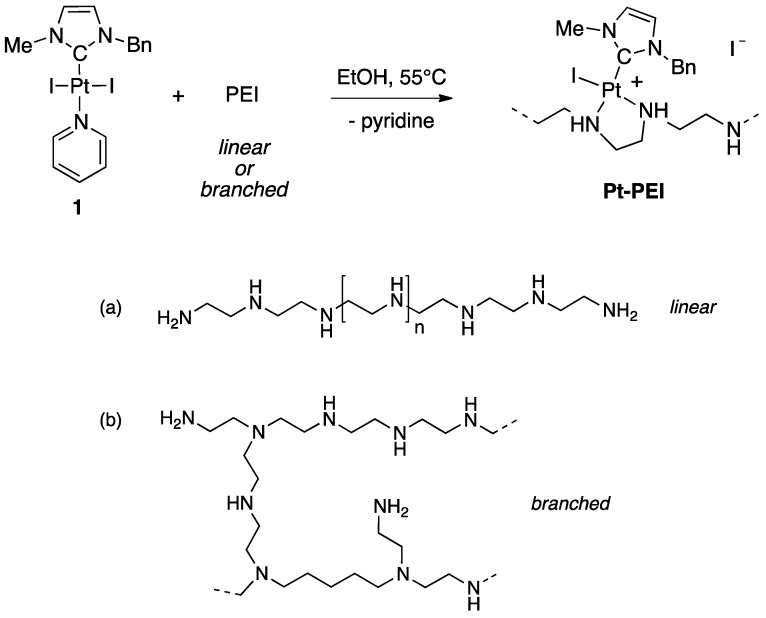
Synthesis of NHC-Pt-PEI conjugates prepared from *trans* [(NHC)PtI_2_(pyridine)] (1) (NHC = 3-benzyl-1-imidazolilydene) (EtOH, 55 °C, 48 h, quantitative). Chemical structure of (**a**) linear and (**b**) branched PEI.

Ligand substitution on a preformed NHC-platinum pyridine complex is a direct and practical method for the synthesis of conjugates [11,12]. This strategy allowed us to easily generate libraries of functionalized Pt(II)-NHC complexes [13]. Using the same strategy, NHC-platinum-PEI conjugates were synthesized by reacting *trans* [(NHC)PtI_2_(pyridine)] (1) (NHC = 3-benzyl-1-imidazolilydene) and the desired PEI polymer chain (Figure 2). In very mild condition (EtOH, 55 °C), the complete grafting of the NHC platinum complexes on polyethyleneimine (PEI) was observed after 48 h of reaction. The conversion was quantitative and variation in the nature (linear or branched) and in the molecular weight of the PEI as well as the ratio between the number of platinum centres and the number of nitrogen atoms gave access to a large variety of polycationic NHC-Pt-PEI conjugates with different metal-loadings. The grafting of the NHC-Pt moiety was easily evidenced by NMR spectroscopy thanks to the characteristic peaks of the NHC ligand and to the disappearance of the pyridine signals. Moreover, integration of the characteristic signals of the NHC ligand by ^1^H NMR spectroscopy allowed us to determine the Pt:NH ratio of the NHC-Pt-PEI conjugates that were found to be in accordance with the Pt- and N-quantification obtained by elementary analyses.

Four different polyethylenimines were used: a branched polymer of 1.8 kDa and three linear polymers of 2.5, 25 and 250 kDa. The ratio between the number of platinum centres and the number of nitrogen atoms was also varied, thus affording NHC-Pt-PEI conjugates with various metal-loadings. Interestingly, all NHC-Pt complexes showed an improved water-solubility. Whereas stock solutions in DMSO are generally required to solubilize NHC-metal complexes, NHC-Pt-PEI conjugates are easily solubilized in ethanol and then could be diluted in aqueous solutions such as cell culture medium.

#### 2.1.2. Fluorophore-Tagged NHC-Pt-PEI Conjugates

**Figure 3:**
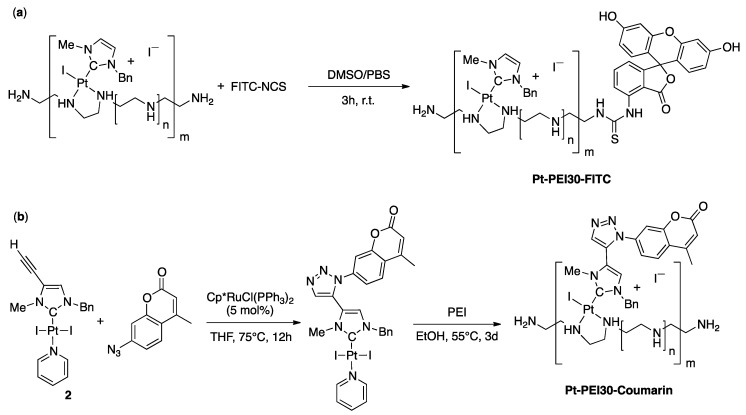
Synthesis of FITC-labelled Pt-PEI30 Pt-PEI30-FITC (**a**) and direct functionalization of the NHC platinum complex 2 prior to synthesis of the NHC-Pt-PEI conjugate Pt-PEI30-Coumarin (**b**).

For monitoring the uptake and intracellular distribution of the NHC-Pt-PEI conjugates, two fluorophore-tagged analogues of the conjugate were synthesized. First, the Pt-PEI30 conjugate was labelled with fluorescein isothiocyanate FITC (Figure 3a) which is well known for studying biological systems and is suitable for in vitro fluorescence microscopy imaging. The reaction of one primary amine at the extremity of the PEI chain with the fluorescein isothiocyanate spontaneously occurred within three hours in a 1/2 mixture of DMSO/PBS at room temperature. Subsequent dialysis over large volume of PBS for one day followed by a lyophilization quantitatively yielded the expected FITC-labelled conjugate Pt-PEI30-FITC. Alternatively, a parallel synthetic route involving PEI label with fluorescein prior to platinum coordination has been developed and allowed successful isolation of the same conjugate and is expected to allow further development under milder conditions using more sensitive NHC-Pt motifs. Secondly, coumarin which is a UV-excitable fluorophore emitting fluorescence at a different wavelength from FITC, was directly attached to the Pt-NHC complex by covalent functionalization using alkyne-azide cycloaddition reaction [14,15]. The reaction was carried out using a catalytic amount of [Cp*RuCl(PPh_3_)_2_] with the alkyne-functionalized [(NHC)PtI_2_(pyridine)] derivative (2) and two equivalents of 7-azido-4-methylcoumarin in dry THF (Figure 3b). Complexation of the coumarin-functionalized NHC-Pt moiety has then been achieved using a 1Pt/30NH ratio and three days under dark conditions proved necessary for full platinum precursor consumption and quantitative recovery of NHC-Pt-PEI conjugate by centrifugation.

### 2.2. In Vitro Study

#### 2.2.1. Polymer Size Effect

**Figure 4:**
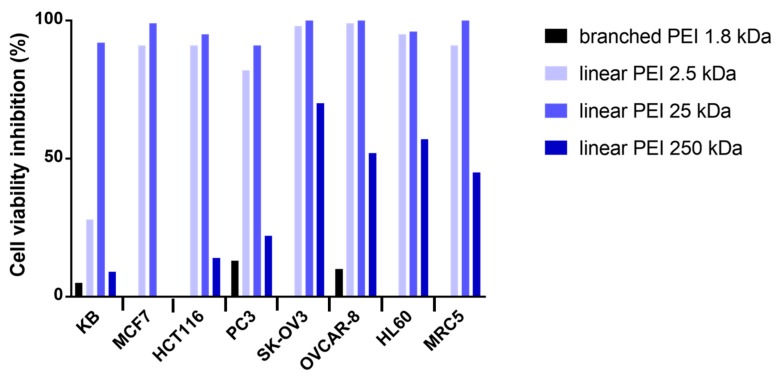
Effects on different cell lines of NHC-Pt-PEI conjugates at concentration of 10^−5^ M (platinum concentration) with branched PEI 1.8 kDa (black), linear PEI 2.5 kDa (light blue), linear PEI 25 kDa (blue) and linear PEI 250 kDa (dark blue). A fixed ratio of 20 was used between Pt and monomeric PEI units.

The cytotoxicity of the NHC-Pt-PEI conjugates was investigated on a panel of 7 cancer cell lines, namely, KB (epidermal carcinoma), MCF7 (breast adenocarcinoma), HCT116 (human colorectal adenocarcinoma), PC3 (human prostate adenocarcinoma), SK-OV3 (ovarian adenocarcinoma), OVCAR-8 (human ovarian carcinoma), HL60 (acute promyelocytic leukaemia) and one cell line derived from normal lung tissue (MRC5). The aim of the study was first to evaluate the polymer size effect while keeping the same platinum/polymer unit ratio. Thus, four NHC-Pt-PEI conjugates that contain one Pt complex per 20 ethylene diamine units were investigated, one with a branched PEI 1.8 kDa and 3 with linear PEI (2.5, 25 et 250 kDa). The toxic effects were evaluated by measuring cell metabolic activity using 3-(4,5-dimethylthiazol-2-yl)-5-(3-carboxymethoxy-phenyl)-2-(4-sulfophenyl)-2H-tetrazolium (MTS). The results are shown in Figure 4.

As shown in Figure 4, NHC-Pt-PEI conjugate containing branched PEI gave essentially no cytotoxic activity whereas the conjugates with linear PEI displayed a promising cytotoxicity against all cell lines at a concentration of 100 µM (platinum concentration). While looking at the linear macromolecule size effect, at a concentration of 100 μM, the best activity was observed with 2.5 and 25 kDa PEI (92–100% cell viability inhibition). However, the conjugate with the largest PEI (250 kDa) showed poor cell viability inhibition at this concentration. To continue the studies, we decided to focus on 25 kDa PEI as it displayed a good cytotoxic activity and as it is close to 22 kDa PEI, which is well known for its transfection ability [16].

#### 2.2.2. Platinum Concentration Effect

**Figure 5:**
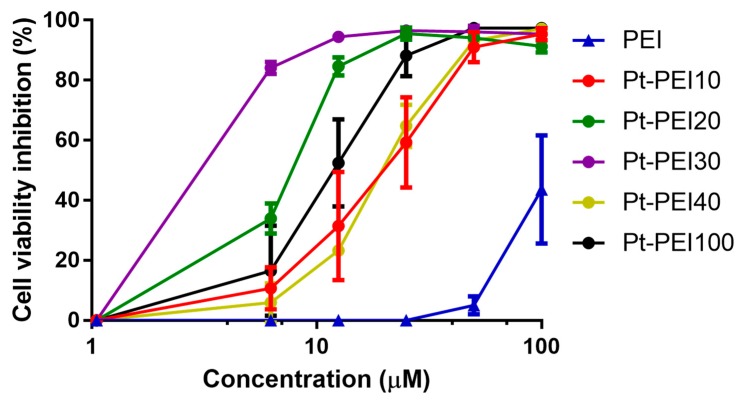
Effects on HCT116 cell metabolic activity of NHC-Pt-PEI conjugates with various metal ions density onto polymer chains. The numbers refer to the ratio between monomeric PEI units and platinum ions (Pt/NH). Results are expressed as mean ± SEM of at least three independent experiments. All concentrations (in μM) are expressed as platinum concentration except for PEI.

The metal complex/polymer ratio was varied in order to obtain NHC-Pt-PEI conjugates that contain one platinum complex per 10, 20, 30, 40 or 100 ethylenediamine units (referred to as Pt-PEI10, Pt-PEI20, etc.). These ratios correspond to ca. 60, 30, 20, 15 and 6 platinum atoms per PEI chain of 25 kDa, respectively. The cytotoxicity of these conjugates was then investigated on human colorectal adenocarcinoma cells (HCT116) as shown in Figure 5 (all concentrations are expressed as function of platinum concentration and not polymer concentration) [17]. The toxic effects were evaluated by measuring cell metabolic activity using MTS after 24 h of treatment. Activity was dependent on the density of metal ions on the polymer chain.

As shown in Figure 5, all the NHC-Pt-PEI conjugates display a strong cytotoxic activity against HCT116 cells. Nevertheless, the NHC-Pt-PEI compound containing one platinum complex per 30 ethylenediamine units, namely Pt-PEI30, has the smallest IC_50_ value. That is why we decided to continue our studies with this compound. The stability in solution of Pt-PEI30 has been investigated and no change of the activity (in vitro) was observed while keeping the conjugate Pt-PEI30 in ethanol solution for more than 2 months at 4 °C.

### 2.3. Short Time Exposure with NHC-Pt-PEI30 is Enough to Induce High Cytotoxicity Activity

**Figure 6:**
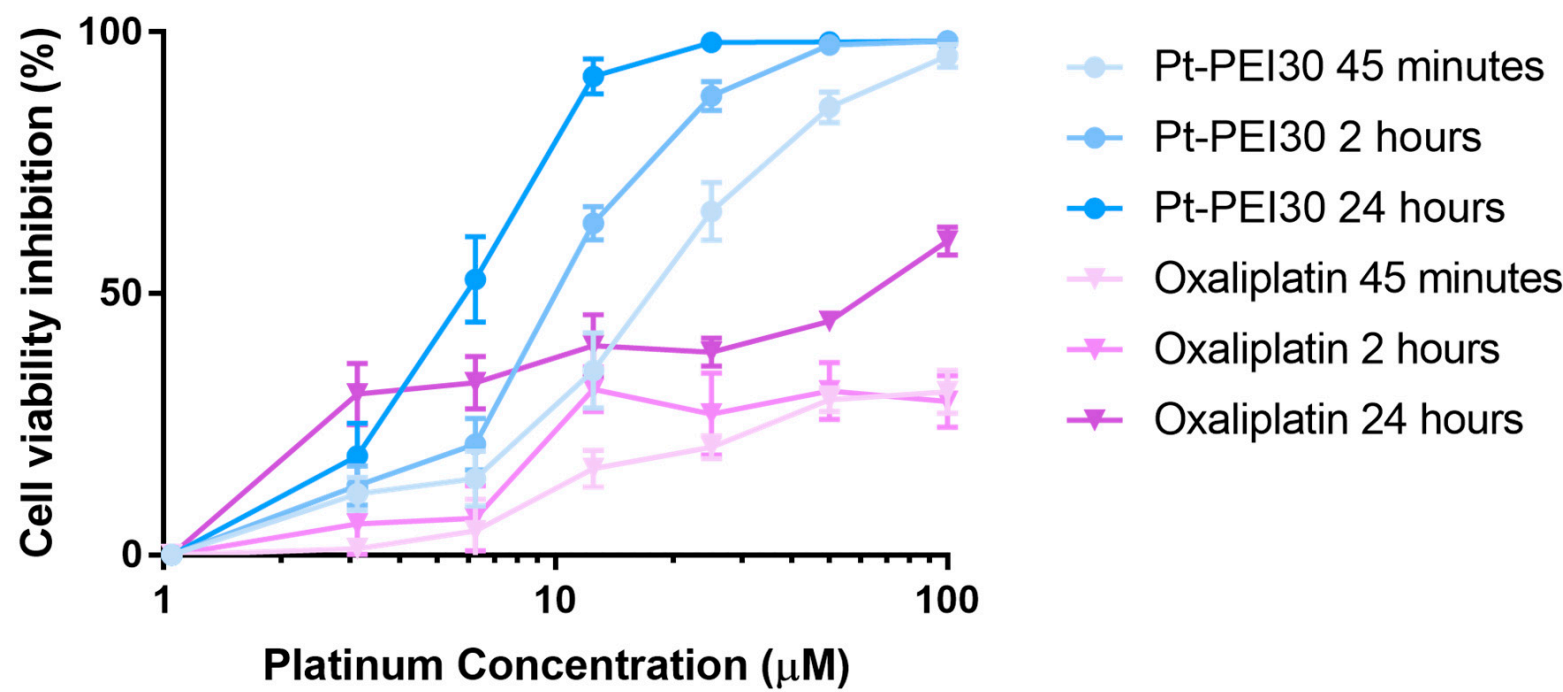
Effects on HCT116 cell metabolic activity of various exposure times of Pt-PEI30 conjugate and oxaliplatin as a reference. Results are expressed as mean ± SEM of at least four independent experiments. All concentrations (in μM) are expressed as platinum concentration.

To go further in NHC-Pt-PEI conjugate mode of action, we evaluated the time exposure sufficient to promote in vitro cell cytotoxicity. For this, HCT116 cells were incubated with Pt-PEI30 for 45 min and 2 h, then the cytotoxic platinum compound was removed and replaced by cell culture medium. Cell cytotoxicity was evaluated 24 h after exposure by measuring cell metabolic activity using MTS. Incubation of cells with Pt-PEI30 during the whole 24 h was used as control assay. As shown in Figure 6, a 45 min exposure is sufficient to induce a high cytotoxic activity in the same range as the one induced after 24 h of incubation. These results suggest a quick uptake of the NHC-Pt-PEI conjugate by the cells. Oxaliplatin was used as a reference in our experiment (Figure 6) and the data showed that it required longer time exposure for efficacy.

### 2.4. Cellular Uptake of Pt-PEI30 

**Figure 7:**
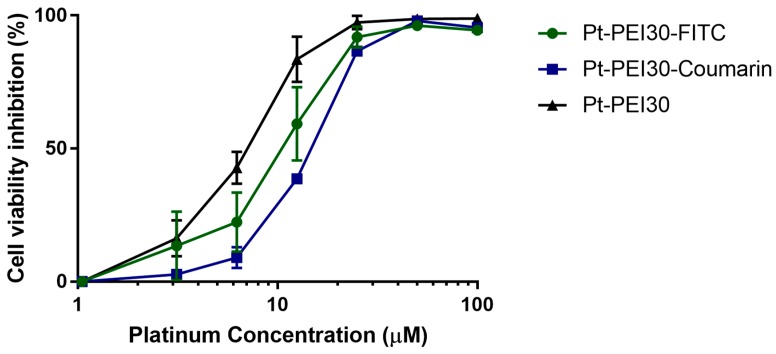
Effects on HCT116 cell metabolic activity of fluorescent Pt-PEI30 conjugates and non-fluorescent Pt-PEI30 compound as a reference. Results are expressed as mean ± SEM of at least two independent experiments. All concentrations (in μM) are expressed as platinum concentration.

To follow cellular uptake of NHC-Pt-PEI30 conjugate, two fluorescent derivatives were used. The fluorophore was either attached onto the polymer chain (i.e., Pt-PEI30-FITC) or directly onto the *N*-Heterocyclic carbene (i.e., Pt-PEI30-Coumarin). The impact of the fluorescent probe on the cytotoxic activity of the conjugate was first investigated by measuring cell metabolic activity of HCT116 cells after 24 h of exposure. As depicted in Figure 7, both displayed a slight and non-significative decrease of activity which is negligible compared to the overall activity.

**Figure 8:**
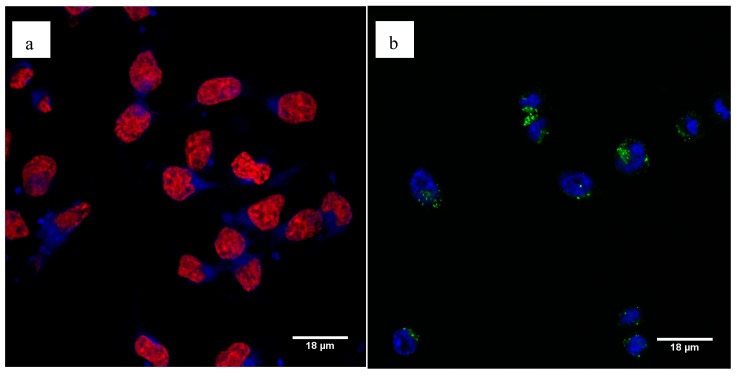
Cellular uptake of fluorescent Pt-PEI30 compounds was visualized by confocal microscopy after 3h30 treatment on HCT116 cells. (**a**) Cells were treated with Pt-PEI30-Coumarin conjugate (in blue) and cell nucleus was stained using DRAQ5 (in red), (**b**) cells were treated with Pt-PEI30-FITC conjugate (in green) and cell nucleus was stained with Hoechst 33342 (in blue).

Using confocal microscopy, we showed efficient cell uptake of the fluorescent Pt-PEI30 conjugates. We observed the fluorescence with the two conjugates showing that attachment of the fluorophore directly onto the *N*-Heterocyclic carbene complex (i.e., Pt-PEI30-Coumarin, a) or onto the PEI polymer chain (i.e., Pt-PEI30-FITC, b) has no effect on their activity. The additional staining of the cell nucleus either with DRAQ5 for the Pt-PEI30-Coumarin compound (Figure 8a) or with Hoechst 33342 for the Pt-PEI30-FITC compound (Figure 8b) showed that fluorescent Pt-PEI30 conjugates were essentially found in the cytosol and only at a very low extent in the nucleus. These observations suggest that the conjugates enter the cell through endocytosis. Moreover, the localization in the cytosol and not in the nucleus correlated with a cytotoxicity independent of nucleus events.

## 3. Discussion

Numerous strategies have been studied in order to circumvent side effects associated with cisplatin-based chemotherapy. In particular, drug delivery based on polymeric assemblies has raised massive interest to protect the platinum centre from side reactions and to promote selective Pt accumulation into the cancer cells [18]. Polyethyleneimine (PEI) is well established as polycationic, water-soluble and biocompatible carrier, especially for transfection purpose [19,20]. In vivo, such polycationic macromolecules are expected to target cells thanks to electrostatic interactions with the negatively charged phospholipid cellular membrane. Drug delivery is believed to occur through cell endocytosis of the drug-PEI assembly. Endosomal escape is favoured by the action of enzymes (ATPase) which acidify the media within the particle thus promoting PEI protonation and elongation. Subsequent osmotic swelling provokes a rupture of the endosomal membrane and drug release into the cytoplasm [21].

We synthesized platinum *N*-Heterocyclic carbene bioconjugates by using polyethyleneimine as polymer carrier. In these systems, branched and linear PEI polymers with various sizes were used as ligand to stabilize the *N*-Heterocyclic platinum carbene complexes. Interestingly, the introduction of PEI ligand to the Pt-NHC fragment induced good water-solubility of the overall system. Whereas stock solutions in DMSO are generally required to solubilize classical NHC-metal complexes, these NHC-Pt-PEI conjugates are solubilized in ethanol at a concentration of 5 mM and then diluted in aqueous solutions for further biological studies (final concentration of EtOH at 10 μM = 0.2%).

Investigation of the biological activities on several cancer cell lines showed that Pt-PEI with branched polymer were ineffective whereas Pt-PEI with linear polymer of 2.5 or 25 kDa displayed very good activities at concentration of platinum metal down to 10^−5^ M. Since the cell viability inhibitions were slightly higher with a PEI of 25 kDa, we selected this size of polymer for further investigations. Interestingly, the highest efficiencies for gene delivery both in vitro and in vivo are also obtained with PEI of 25 kDa [22,23]. Next, biological activity as function of the overall density of metal ions onto 25 kDa PEI polymer chain revealed an optimal ratio Pt/PEI unit of 30 (IC_50_ of ~3 μM). We conclude that the conjugate Pt-PEI30 with linear PEI of 25 kDa is the best candidate for further development as anticancer agent.

Pt-PEI30 exhibits a higher cytotoxic activity than oxaliplatin at the same platinum concentration. Moreover, its cytotoxic activity is induced after very short exposures of the cells suggesting a rapid uptake of the conjugate by the cells, sufficient to induce pathways leading to death. In a previous study, we showed that Pt-PEI30, in contrast to oxaliplatin, induced cell apoptosis by a mechanism, at least partly, independent of the nucleus [10]. As mitochondria dysfunctions were induced by the compound, we suggest that apoptosis was induced by the mitochondrial pathway [24].

To go further in the Pt-PEI30 mode of action, we tracked the fate of Pt-PEI30 in the cells. For this, we synthesized two types of fluorescent-tagged conjugates: one carrying the fluorophore on the polymer and the other directly on the *N*-Heterocyclic carbene. The two fluorescent conjugates displayed a high cytotoxic activity and strong fluorescence capacity. Interestingly, pictures with the two fluorescent conjugates showed the same features: multiple dots in the cytosol and very few in the nucleus. These features are totally in accordance with the hypothesis of the cell death induction through a mitochondrial pathway. Moreover, as the pictures of cells incubated with the two fluorescent Pt-PEI30 derivatives are similar, we can hypothesize that PEI and NHC remains tethered after cell uptake at least during the 3 first hours of the experiment. The next step will consist of a kinetic analysis of the localization of the two moieties (PEI and NHC) in the cell and more particularly in the different organelles like the mitochondria. This precise analysis will permit to better understand the Pt-PEI30 mode of action and then, to optimize the design of conjugates for a better anticancer activity.

## 4. Materials and Methods

### 4.1. General Remarks

All manipulations of air and moisture sensitive compounds were carried out using standard Schlenk techniques under an inert atmosphere of argon and solvents were purified and degassed following standard procedures. All reagents were purchased from commercial chemical suppliers (Acros Organics, Illkirch, France; Alfa Aesar, Karlsruhe, Germany and TCI Europe, Paris, France) and used without further purification. ^1^H and ^13^C Nuclear Magnetic Resonance (NMR) spectra were recorded on a Bruker Avance 300 or a Bruker Avance 500 spectrometer using the residual solvent peak as a reference (CDCl_3_: δH = 7.26 ppm; δC = 77.16 ppm) at 295 K. The two [(NHC)PtI_2_(pyridine)] precursors (1 and 2) used for the synthesis of Pt-PEI, Pt-PEI30-FITC or Pt-PEI30-coumarin were synthesized according to reported procedures [10,15].

### 4.2. Synthesis of NHC-Pt-PEI Complexes

NHC-Pt-PEI: Pt-PEI30. Under argon, a solution of *trans* [(NHC)PtI_2_(pyridine)] 1 (NHC = 3-benzyl-1-imidazolilydene) (20 mg, 28.6 μmol) and PEI (36.9 mg, 25 kDa) in ethanol (10 mL) was stirred 2 days at 55 °C. The colour of the solution is turning from yellow to colourless. The solution was concentrated under reduced pressure, precipitated by addition of excess diethyl ether and subsequently washed to afford the Pt-PEI30 as a white solid (55 mg, quant.). The Pt/ethylenediamine unit ratio was confirmed by elemental analysis and ^1^H NMR. Anal. Calcd for [(PEI 25 kDa)(C_11_H_12_N_2_PtI_2_)_20_]: C, 44.3; H, 8.4; N, 23.2; Pt, 10.4; Found: C, 43.6; H, 8.1; N, 22.7; Pt, 9.8. ^1^H-NMR (CD_3_OD/CD_3_CN, 300 MHz, 20 °C): δ 2.2-3.1 (m, CH_2_), 3.1–3.6 (m, NH_2_ and NH), 4.0–4.5 (m, N-CH_3_), 5.9 (m, N-CH_2_), 7.1–8.0 (m, H_ar_ and 2 C-H). The same experimental procedure was used for all NHC-Pt-PEI conjugates.

Pt-PEI30-FITC: To a solution of Pt-PEI30 (20 mg, 0.54 μmol) in DMSO (1 mL) and PBS (2 mL) was added a solution of fluorescein isothiocyanate (0.026 mg, 0.054 μmol) in DMSO (100 μL) and stirred in the dark at 20 °C, for 3 h. The solution was concentrated under reduced pressure, diluted in ethanol (0.3 mL) and subsequently dialyzed toward PBS (500 mL) at 20 °C for 24 h. The mixture was then recovered and lyophilized under reduced pressure to afford the NHC-Pt-PEI conjugate as a deep orange muggy solid. Orange oil, quant. ^1^H NMR (MeOD, 500 MHz, 20 °C): δ 0.81–1.19 (m, CH_2_,PEI), 2.87 (bs, CH_2_,PEI), 3.22 (bs, CH_2_,PEI), 3.25–3.55 (m, CH_2_,PEI), 3.87–4.12 (m, CH_2_,PEI + N-CH_3_), 4.75 (bs, CH_2_,PEI + NHPEI), 5.41–5.90 (m, N-CH_2_), 6.41–6.42 (m, H_ar_), 7.01–8.46 (m, H_ar_); UV-vis (EtOH) λ_max_ (nm): 508; Fluorescence: λ_ex_ = 508 nm, λ_em_ = 525 nm.

Pt-PEI30-coumarin: By adaptation of a reported synthesis [15], a solution of alkyne-functionalized [(NHC)PtI_2_(pyridine)] 2 (6.5 mg, 7.5 × 10^−6^ mol) in THF (1.5 mL) and a solution of 7-azido-4-methylcoumarin (3 mg, 1.49 × 10^−5^ mol) in THF (1 mL) were added to a solution of [RuClCp*(PPh_3_)_2_] (0.6 mg, 7.45 × 10^−7^ mol) in THF (1 mL). The mixture was then heated overnight at 75 °C. The solvent was then removed under vacuum. The residue was purified by means of silica gel chromatography using a mixture of CH_2_Cl_2_/pentane 3:1 followed by CH_2_Cl_2_ and then ethyl acetate to afford the compound as a yellow-brown oil. Under argon, a solution of coumarin functionalized [(NHC)PtI_2_(pyridine)] complex (20 mg, 28.6 μmol) and linear poly(ethyleneimine) of 25 kDa (36.9 mg, 1.47 μmol) in ethanol (10 mL) was stirred for three days at 55 °C. The resulting solution was then concentrated under reduced pressure, precipitated by addition of excess diethyl ether and further centrifuged for 10 min at 10,000 rpm to afford the NHC-Pt-PEI conjugate as a yellow muggy solid. Light yellow muggy solid, quant. ^1^H NMR (MeOD, 500 MHz, 20 °C): δ 0.91 (t, *J* = 6.7 Hz, CH_2_,PEI), 1.12 (t, *J* = 6.7 Hz, CH_2_,PEI), 1.26–1.41 (m, CH_2_,PEI), 2.22 (s, CH_3_), 2.77 (m, CH_2_,PEI), 3.32–3.65 (m, CH_2_,PEI), 3.92 (m, N-CH_3_), 5.08–5.37 (m, CH_2_,PEI + N-CH_2_), 5.50 (bs, CH_2_,PEI + NHPEI), 6.51 (m, CH_im_), 6.62 (m, H_ar_), 6.91 (m, H_ar_), 6.93–7.47 (m, H_ar_), 8.07 (m, H_ar_), 8.56 (s, H_ar_). UV-vis (EtOH) λ_max_ (nm): 370; Fluorescence: λ_ex_ = 370 nm, λ_em_ = 455 nm. 

### 4.3. Preparation of Platinum Derivatives for in Vitro Assays

Oxaliplatin was purchased from Sigma Aldrich, (Saint-Louis, MO, USA) and was prepared by dissolution in H_2_O at 5 mM (metal concentration). NHC-Pt-PEI conjugates PEI10, PEI20, PEI30, PEI40, PEI100, PEI30-FITC and PEI30-coumarin stock solutions were prepared by dissolution in absolute ethanol at 5 mM (metal concentration). Samples were then diluted in cell culture medium (RPMI 1640 supplemented with 10% (*v*/*v*) of heat-decomplemented foetal calf serum and Penicillin-Streptomycin (10U–0.1 mg/mL)) referred as complete medium.

### 4.4. Cell Exposure to the Different Platinum Compounds for Cell Viability Assays

Human cancer cell line HCT116 (colorectal adenocarcinoma) was cultured in complete medium at 37 °C with 5% CO_2_, 80% humidity. For the cell viability experiments, cells were seeded in 96-well plates at 3 × 10^4^ cells per well in 50 µL of complete medium. After they had adhered to the culture plate, the cells were exposed to the different platinum compounds (NHC-Pt-PEI or oxaliplatin as control) during 45 min, 2 or 24 h at 37 °C (5% CO_2_). For the treatments of 45 min and 2 h, cells were exposed to the platinum derivatives for these times and then the treatment was replaced by new complete culture medium until 24 h.

### 4.5. Evaluation of Cell Viability by MTS Assays

After 24 h, 20 μL of the tetrazolium compound 3-(4,5-dimethylthiazol-2-yl) -5-(3-carboxymethoxyphenyl)-2-(4-sulfophenyl)-2H-tetrazolium (MTS) (Promega Corporation, Madison, WI, USA) were added in each well. After 2 h of incubation at 37 °C, absorbance was measured at 490 nm (SP200, Safas, Monaco), which is directly proportional to the number of metabolically active living cells in culture. The absorbance of the blank (RPMI + MTS) was subtracted from the values of each well and the optical density (OD) of non-treated cells was considered as 100% of viability. The percentage of cell viability inhibition was calculated using the following formula: % cell viability inhibition = 100 − [OD (treatment)/OD (100% viability) × 100].

### 4.6. Cell Exposure to the Fluorescent Platinum Compounds and Confocal Microscopic Analysis

Cells were seeded on coverslips disposed in a 24-well plate at 7.5 × 10^4^ cells per well in 500 µL of complete medium and maintained at 37 °C (5% CO_2_) overnight for adherence. Then, the culture medium was removed and replaced by the platinum conjugates (6.25 µM platinum concentration) for 3 h 30. After several washing steps, the cells were fixed in paraformaldehyde 1%. The nucleus was stained either with Hoechst 33342 (1 µg/mL, Sigma-Aldrich, Saint-Quentin Fallavier, France) or with DRAQ5 (5 µM, Abcam, Cambridge, UK) and coverslips were mounted on slides using ProLong Gold Antifade Reagent (Life Technologies, Waltham, MA, USA) and visualized by confocal microscopy (Leica TSC SPE, Wetzlar, Germany).
